# A Novel SEM Image Processing Approach for Evaluating Sterilization Effects on Polymeric Medical Devices: Validation Against Traditional EDX Analysis

**DOI:** 10.3390/polym17233156

**Published:** 2025-11-27

**Authors:** Mohamed A. Aboamer, Rashed Almousa, Ahmad Alassaf, Abdulrahman Alduraywish, Ibrahim AlMohimeed, Talal Alharbi, Vidan F. Ghoneim

**Affiliations:** 1 Department of Medical Equipment Technology, College of Applied Medical Sciences, Majmaah University, Majmaah 11952, Saudi Arabia; ra.almousa@mu.edu.sa (R.A.); am.alassaf@mu.edu.sa (A.A.); a.aldurywish@mu.edu.sa (A.A.); i.almohimeed@mu.edu.sa (I.A.); 2 Department of Electrical Engineering, College of Engineering, Qassim University, Buraydah 52531, Saudi Arabia; atalal@qu.edu.sa; 3 Department of Biomedical Engineering, College of Engineering, Princess Nourah bint Abdulrahman University, P.O. Box 84428, Riyadh 11671, Saudi Arabia; vfghoneim@pnu.edu.sa

**Keywords:** SEM image processing, EDX validation, UVC sterilization, ABS polymer, statistical analysis

## Abstract

This study aimed to evaluate the impact of UVC (Ultraviolet C Radiation), detergent foam, and alcohol (70%) sterilization methods on the surface morphology of acrylonitrile–butadiene–styrene (ABS) specimens using a novel SEM (Scanning Electron Microscope) image processing approach. Twelve 3D-printed specimens were prepared, and five concentric circular regions of interest (ROIs) per specimen were analyzed. Three quantitative descriptors—defect area fraction, anisotropy ratio, and RMS (Root Mean Square) roughness—were extracted to assess surface alterations. To validate the image-based findings, EDX (Energy-Dispersive X-ray Spectroscopy) elemental analysis for carbon (C), nitrogen (N), and oxygen (O) was employed as a complementary and traditional benchmark technique. Statistical comparisons and *p*-value heat maps revealed strong convergence between SEM and EDX results. UVC sterilization consistently preserved surface morphology and elemental stability, showing the lowest defect fraction (*p* = 0.2684), balanced anisotropy (*p* = 0.02481), and minimal oxygen incorporation (O = 7.6). Foam sterilization produced intermediate effects, with significant anisotropy changes (*p* = 0.007456) and reduced nitrogen (19.6). Alcohol sterilization induced the most severe damage, characterized by high defect density, increased roughness, and elemental imbalance (N = 17.3, O = 13.9), confirming oxidative degradation. The convergence of SEM and EDX outcomes demonstrates that SEM image processing is a reliable novel method validated by traditional elemental analysis. Together, these approaches provide a robust framework for ranking sterilization efficacy, with UVC identified as the most favorable method, detergent foam as an acceptable alternative, and alcohol as the least effective due to its destabilizing effects.

## 1. Introduction

Healthcare-associated infections (HAIs) represent a critical global health challenge, contributing substantially to morbidity, mortality, and escalating healthcare costs. The World Health Organization (WHO) estimates that hundreds of millions of patients are affected annually [1]. Effective disinfection of medical device surfaces is therefore essential to mitigate microbial transmission in healthcare facilities.

Acrylonitrile–butadiene–styrene (ABS) is one of the most widely used medical-grade polymers due to its strength, chemical resistance, and ease of processing [2]. It can be manufactured by injection molding or 3D printing into complex shapes with smooth surfaces, making it suitable for housing, frames, connectors, and protective cases in medical devices [2]. Recent studies have demonstrated that prolonged UVC sterilization can significantly alter the mechanical properties of 3D-printed ABS parts, underscoring the need to investigate how different disinfection methods affect this material in medical applications [3].

Chemical disinfectants remain the cornerstone of hospital surface decontamination. Alcohols, particularly 70% ethanol and isopropanol, are widely employed for their broad-spectrum antimicrobial activity, although they lack sporicidal efficacy and may degrade polymers upon repeated application [4]. In recent years, “no-touch” automated disinfection technologies, particularly UVC irradiation, have been introduced as alternatives or complements to manual approaches [5].

UVC-based systems provide rapid, chemical-free disinfection and may reduce reliance on manual cleaning protocols [6]. Comparative investigations show that although both 70% isopropyl alcohol wipes and UVC irradiation effectively reduce microbial contamination, alcohol wipes often demonstrate slightly greater efficacy [7]. Detergent-based foams are also applied in healthcare environments, yet direct head-to-head comparisons between alcohol, UVC, and detergent foams on medical device surfaces remain limited [8]. Clinical evaluations indicate that pulsed-xenon UVC systems can markedly reduce bacterial contamination on high-touch hospital surfaces, frequently outperforming manual cleaning protocols [9]. Moreover, handheld UVC LED devices have demonstrated strong ability to reduce microbial burden near operative areas, achieving >90% decreases in colony-forming units compared to manual cleaning alone [10]. Despite these advances, a distinct gap remains in structural investigations—particularly those using scanning electron microscopy (SEM)—to determine how disinfection methods physically influence material surfaces.

SEM has been extensively used in dentistry and materials science to evaluate surface alterations induced by disinfectants. For instance, Basavarajappa et al. reported that ethanol exposure increased roughness and induced micromechanical changes in denture base polymers [11]. However, no study has systematically applied SEM or energy-dispersive X-ray spectroscopy (EDX) to ABS medical device surfaces after alcohol-, UVC-, or detergent-based disinfection. Established standards such as ISO 22309 [12], Newbury’s electron interaction analysis [13], ASTM E1508 [14], and the microanalysis principles of Goldstein et al. [15] provide an essential framework for reliable SEM–EDX investigations.

Recent advances in image-based analysis have expanded SEM beyond qualitative imaging, enabling the introduction of quantitative surface descriptors. Among these, Defect Area Fraction quantifies the proportion of surface occupied by pores or cracks [16]. The Anisotropy Ratio reflects the directional dependency of material properties, including strength and fatigue resistance, which vary significantly with structural orientation [17]. For example, structural anisotropy in clay shale strongly influences strength and failure, while microstructural anisotropy in additively manufactured alloys correlates with fatigue crack growth [17]. Root Mean Square (RMS) Roughness captures nano- to microscale variations in surface height and can be derived from properly processed SEM images calibrated against atomic force microscopy (AFM), as demonstrated by [18]. These descriptors provide robust metrics for linking disinfection-induced morphological changes to functional performance.

Building on prior research that investigated ABS degradation under prolonged UVC sterilization [3], which primarily focused on bulk mechanical properties without addressing surface-level changes; polymer surface alterations following ethanol disinfection [11], which examined only dental polymers under a single chemical disinfectant; and the application of quantitative SEM descriptors for microparticle roughness analysis [18], which was limited to particle-scale systems rather than polymeric medical devices, the present study provides the first systematic assessment of ABS medical device surfaces exposed to alcohol, UVC, and detergent foam disinfection. By introducing standardized specimen preparation, structured region-of-interest (ROI) segmentation for SEM image analysis, and the application of quantitative descriptors—including Defect Area Fraction, Anisotropy Ratio, and RMS Roughness—this work aims to deliver reproducible insights into both the safety and structural integrity of ABS under clinically relevant disinfection protocols.

### 1.1. Problem Question

How do different sterilization methods (UVC, detergent foam, and alcohol) affect the surface morphology and elemental composition of materials, and can EDX analysis effectively validate SEM image processing results to identify the most reliable sterilization technique?

### 1.2. Objectives

To evaluate the impact of UVC, detergent foam, and alcohol sterilization methods on surface morphology using SEM-derived parameters: defect area fraction, anisotropy ratio, and RMS roughness.

To analyze the elemental composition (C, N, O) of sterilized surfaces using EDX in order to confirm and validate the morphological changes detected through SEM image processing.

To compare and rank the sterilization methods based on combined SEM and EDX findings and to identify the method that best preserves structural and elemental stability.

## 2. Materials and Methods

### 2.1. Proposed Approach

In this study, twelve cylindrical ABS specimens (diameter ≈ 4 mm, thickness ≈ 3 mm) were fabricated using a 3D Printer, Bambu Lab. H2D: Shenzhen, China, with 75% infill and a gyroid structure, designed to fit standard SEM stubs. The samples were divided into four groups (each group has three samples): untreated control, alcohol (70% ethanol), detergent disinfection foam, and UVC, with each treatment applied for 30 min under controlled conditions. After treatment, all samples were examined by scanning electron microscopy (SEM), JSM-6360, Tokyo, Japan, using identical parameters (acceleration voltage 20 kV, magnification 500×, working distance 11 mm, spot size 30).

For each SEM micrograph, five concentric circular regions of interest (ROIs) with diameters of 4, 6, 8, 10, and 12 cm (scaled at 500×) were extracted to ensure consistent feature sampling and increase the number of samples to 15 in each group where a limited number of samples is common and scientifically accepted in polymer surface characterization [19]. Three surface descriptors were quantified: (i) defect area fraction, calculated as the ratio of pores or cracks to total ROI area; (ii) anisotropy ratio, derived from orientation analysis of surface features to reflect directional dependence linked to strength, failure, and fatigue; and (iii) RMS roughness, computed from calibrated grayscale intensities to capture nano- to microscale height variations. Each descriptor was averaged across ROIs to yield sample-level metrics.

**Figure 1:**
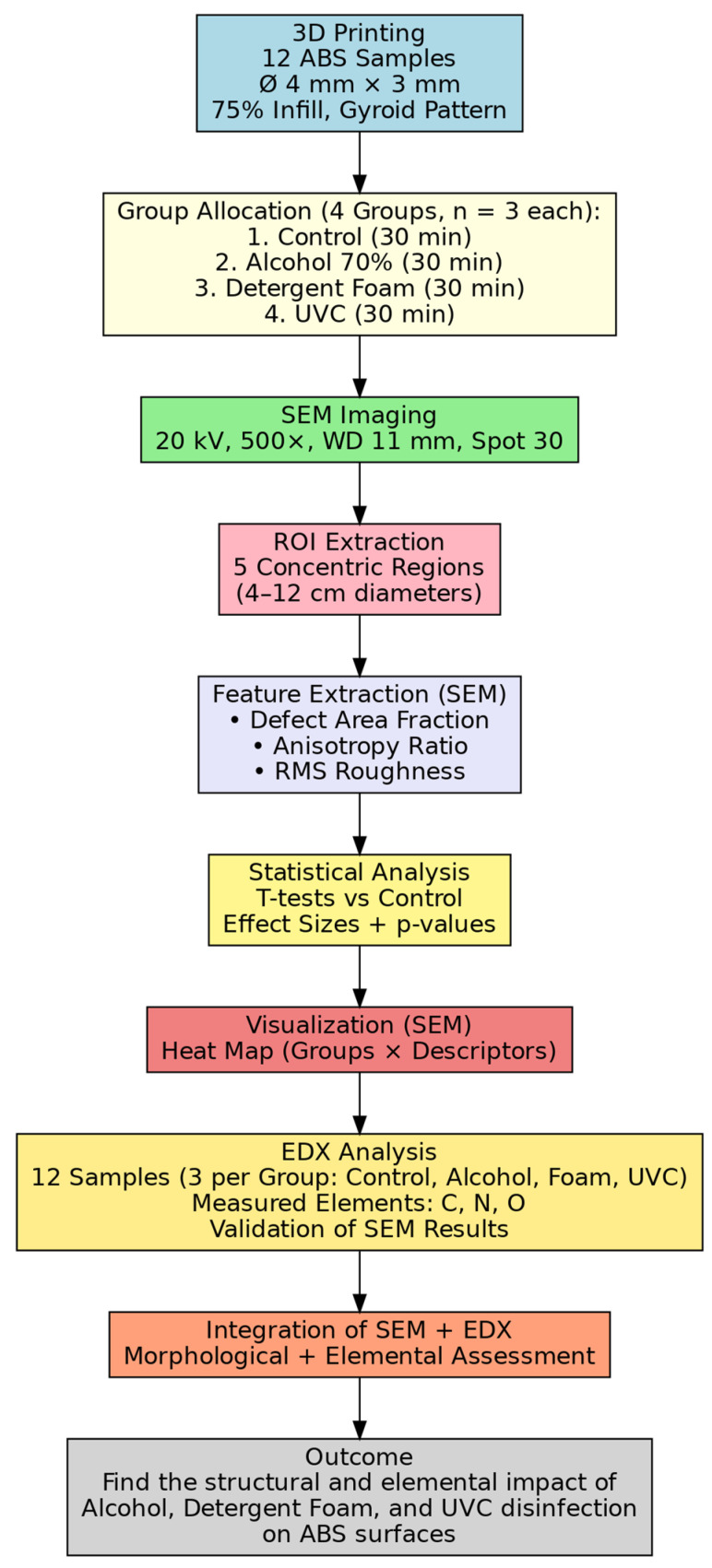
Workflow of the Proposed Experimental Approach for Evaluating the Structural Impact of Alcohol, Detergent Foam, and UVC Disinfection on ABS Surfaces.

Statistical comparisons were performed using two-sample *t*-tests between each treatment group and the control, with effect sizes reported alongside *p*-values. To facilitate interpretation, results were visualized in a heat map with treatments on the *x*-axis and descriptors on the *y*-axis, color-coded by effect size and annotated with significance levels. This integrated approach ensures reproducible specimen preparation, uniform SEM imaging, quantitative surface characterization, and decision-oriented statistical outputs to evaluate the structural impact of alcohol, detergent foam, and UVC disinfection on ABS medical device surfaces (Figure 1).

To validate the novel SEM image processing technique, the elemental composition of sterilized surfaces was assessed using energy-dispersive X-ray spectroscopy (EDX), focusing on carbon (C), nitrogen (N), and oxygen (O). The integration of SEM (morphological quantification) and EDX (chemical validation) provides a comprehensive framework for assessing structural and elemental changes in ABS surfaces exposed to alcohol, UVC, and detergent foam disinfection.

### 2.2. Fabrication of ABS Specimens Using 3D Printing

Twelve cylindrical ABS specimens (diameter ≈ 4 mm, thickness ≈ 3 mm) were fabricated using a 3D printer with 75% infill and a gyroid structure, tailored to fit standard SEM stubs. The specimens were produced on a Bambu Lab H2D 3D printer [20], equipped with a 0.4 mm brass nozzle. The extrusion process was performed at a nozzle temperature of 240 °C, with the heated bed maintained at 100 °C to ensure proper adhesion of the ABS material and minimize warping. A build plate temperature gradient and a controlled enclosure environment were employed to stabilize thermal conditions during printing. The layer height was set to 0.2 mm, and the print speed was maintained at 50 mm/s—parameters optimized to achieve dimensional accuracy and consistent surface quality for subsequent SEM analysis.

### 2.3. Group Allocation

**Figure 2:**
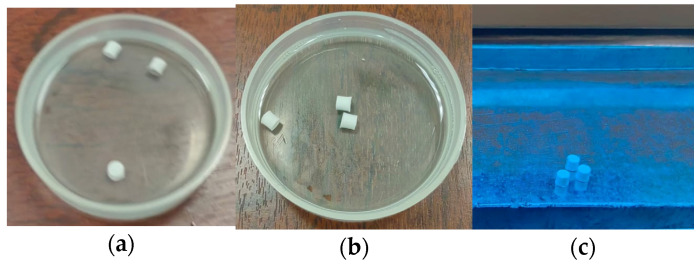
Disinfection process for each group (**a**) alcohol (70% ethanol), (**b**) detergent disinfection foam, and (**c**) UVC.

The samples were divided into four groups (n = 3 per group): a control group, alcohol (70% ethanol) [21], a detergent disinfection foam, and UVC. All treatments were applied for 30 min under controlled conditions, as shown in Figure 2. The detergent foam used was Surfa’Safe Premium (Anios Laboratories, Lille, France), a surface disinfectant formulated for medical-use surfaces [22]. The UVC treatment was performed using two 20 W ultraviolet lamps emitting at 254 nm [22,23,24,25].

### 2.4. SEM Imaging

After treatment, all samples were cleaned only with compressed air to minimize contamination and preserve the surface, before being examined by EDX and SEM using a scanning electron microscope (SEM), JSM-6360, Tokyo, Japan [26] under standardized imaging conditions. The acceleration voltage was set to 20 kV, a value commonly employed for polymeric samples to achieve sufficient beam penetration and generate strong secondary and backscattered electron signals without causing excessive charging or surface damage [27]. A magnification of 500× was selected to visualize surface morphology at the microscale, enabling the detection of pores, cracks, and roughness features while maintaining a representative field of view [28]. The working distance was fixed at 11 mm, providing an optimal balance between depth of field, resolution, and detector efficiency for secondary electron imaging [29]. Finally, a spot size of 30 was applied to ensure a stable electron probe with adequate beam current, balancing signal intensity and resolution to capture quantitative surface features consistently across all samples [30]. These parameters have been widely adopted in SEM investigations of polymers and surface morphology, supporting their appropriateness for the present study.

### 2.5. Region of Interest (ROI) Selection

Four surface conditions were analyzed: untreated Original, UVC disinfected, detergent foam disinfected, and Alcohol disinfected specimens. Each specimen was imaged using scanning electron microscopy (SEM) at fixed magnification to capture surface morphology at a resolution adequate for microstructural analysis. Images were saved in high-resolution PNG format with embedded pixel density metadata (dots per inch, DPI) for scale calibration.

Five concentric circular regions of interest (ROIs) with nominal diameters of 4, 6, 8, 10, and 12 cm were extracted from each SEM image, centered on the geometric center. Pixel scaling was derived from DPI metadata; if absent, a fallback of 37.80 px/cm was applied. The ROI mask *M(x,y)* was defined as [31]

(1) $$M\left(x,y\right)=\left\{\begin{array}{c} 1\;if{\left(x-x_{c}\right)}^{2}+{\left(y-y_{c}\right)}^{2}\;\leq \;r^{2}\;\\ 0\;otherwise\; \end{array}\right.,$$

- *M(x,y)* is the mask function value at pixel coordinates *(x,y)*.
- *M(M(x,y))* is the pixel inside the circular region of interest (ROI).
- M(x,y) is the pixel outside the circular ROI.
- x, y is the pixel coordinates in the image plane.
- Usually, integers index column (x) and row (y) positions of pixels.
- $x_{c}$, $y_{c}$ are the center coordinates of the circle.
- They determine where the circular ROI is positioned in the image.
- r is the radius of the circle, measured in pixels (or converted from physical units if the image scale is known).
- Determines the size of the circular ROI. Larger r → larger circular region included.

The center coordinates (xc, yc) for each specimen were determined automatically using a fixed computational algorithm implemented in MATLAB 2025b. The algorithm first identified the image bounding box then calculated the geometric center of the image, which was subsequently used as the reference point for all concentric ROIs. This fully automated procedure ensured consistent ROI placement across all specimens and eliminated any potential bias associated with manual selection.

### 2.6. Feature Extraction

#### 2.6.1. Defect Area Fraction

##### Count Defect Pixels

- After segmentation, the binary image marks defect pixels as 1 and background as 0.
- The Number of defect pixels counts the number of nonzero elements, i.e., pixels marked as defects [32].

##### Count Total Pixels in ROI

- The circular region of interest (ROI) is defined by the mask.
- The total ROI pixel count is the total number of pixels inside this ROI [32].

##### Compute the Area Fraction

- The defect area fraction (DAF) is the ratio of defect pixels to total ROI pixels [33].

(2) $$DAF=\frac{Number\;of\;defect\;pixels}{Total\;ROI\;pixel},$$

### 2.7. Anisotropy Ratio

#### 2.7.1. Compute Image Gradients

The Sobel operator is used to measure how pixel intensity changes in the x and y directions of the SEM image. These changes are called the image gradients [34].

(3) $$G_{x}=\frac{d_{i}}{d_{x}},\;G_{y}=\frac{d_{i}}{d_{y}},$$

where

- $G_{x}$ is the image gradient in the x-direction (horizontal gradient). It indicates how much the image intensity changes left-to-right.
- $G_{y}$ is the image gradient in the y-direction (vertical gradient). It indicates how much the image intensity changes top-to-bottom.
- $d_{i}$ is the derivative of the image intensity. It means “how intensity changes”—e.g., brightness difference between neighboring pixels.
- $d_{x}$, $d_{y}$ are the partial derivatives with respect to x and y coordinates.
- $\frac{d_{i}}{d_{x}}$ is the change in intensity along the horizontal direction.
- $\frac{d_{i}}{d_{y}}$ is the change in intensity along the vertical direction.

#### 2.7.2. Compute Gradient Magnitude and Orientation [34]

From $G_{x}$ and $G_{y}$ two key values are computed for each pixel:

##### Gradient Magnitude (Edge Strength)

(4) $$G_{m}(x,y)=\sqrt{G_{x}{\left(x,y\right)}^{2}+G_{y}{\left(x,y\right)}^{2}\;},$$

where

- $G_{m}(x,y)$ is the Gradient magnitude at pixel (x,y), meaning the overall edge strength (how strong the brightness change is) at that location.
- $G_{x}(x,y)$ is the Gradient in the x-direction (horizontal) at pixel (x,y), meaning the rate of intensity change left-to-right (detects vertical edges).
- $G_{y}(x,y)$ is the Gradient in the y-direction (vertical) at pixel (x,y), meaning the rate of intensity change top-to-bottom (detects horizontal edges).

##### Gradient Orientation (Edge Direction) [35]

(5) $$\theta (x,y)=\arctan2(-G_{y}(x,y),\;G_{x}(x,y)),\;\theta \;\in \;[0,\pi ],$$

where

- θ(x,y) is the Gradient orientation (direction) at pixel (x,y), meaning the angle of the edge at that pixel, showing which way the intensity changes are pointing.
- θ ∈ [0,π] is the Range of gradient orientations, meaning gradient directions are considered only from 0 to 180° (π radians).

#### 2.7.3. Build the Orientation Histogram

The histogram is defined as

(6) $$H\left(k\right)=\sum_{x,y}G_{m}\left(x,y\right).\;\delta (\left(\theta \left(x,y\right)\in \;{bin}_{k}\right)),\;k=1,\ldots ,N,$$

where

- H(k) is the Orientation histogram value for bin k, meaning the total weighted number of pixels whose edge orientations fall into bin k.
- $G_{m}\left(x,y\right)$ is the Gradient magnitude at pixel (x,y), meaning the strength of the edge at pixel (x,y).
- $\delta (\left(\theta \left(x,y\right)\in \;{bin}_{k}\right))$ is the bin indicator function, meaning

(7) $$\delta \left(\left(\theta \left(x,y\right)\in \;{bin}_{k}\right)\right)=\left\{\begin{array}{c} 1\;if\;orientation\;\theta \left(x,y\right)\;lies\;inside\;bin\;k\\ 0\;otherwise\; \end{array}\right.,$$

This ensures that each pixel contributes only to the correct orientation bin.

- k = 1, …, N is the Orientation bin index, meaning the histogram has N bins that divide the orientation range [0,π].

#### 2.7.4. Normalize the Histogram [36]

- To make the histogram comparable between different images, it is normalized so that the total area = 1:

(8) $$H_{s}\left(k\right)=\frac{H(k)}{\sum_{j=1}^{N}H(i)},$$

where

- H(k) is the histogram value at bin k.
- $\sum_{j=1}^{N}H(i)$ is the total of all bins (the denominator for normalization).

#### 2.7.5. Calculate the Anisotropy Ratio

Finally, anisotropy is defined as how dominant the strongest direction is compared to all others [33,34,35].

(9) $$A=\frac{max(H_{s}(k))}{\frac{1}{N-1}\;\sum_{j\;\neq k_{max}}H_{s}(j)},$$

where

- $max(H_{s}(k))$ is the max of the normalized histogram value for a specific bin k
- $\sum_{j\;\neq k_{max}}H_{s}(j)$ is the means the sum of all the other bins, excluding the maximum one.

### 2.8. RMS Roughness

RMS roughness ($R_{q}$) quantifies surface height variation (approximated from SEM grayscale) [37].

(10) $$R_{q}=\sqrt{\frac{1}{N}\;\sum_{i=1}^{N}(I_{i}-\bar{I}{)}^{2}},$$

- $R_{q}$ is the Root Mean Square (RMS) surface roughness.
- A statistical measure of the average height deviations relative to the mean line (or mean plane in 2D).
- N is the total number of measured data points (pixels, sampling points, or height values) in the profile or surface area.
- $I_{i}$ is the individual height value (or intensity, depending on measurement context) at the ith sampling point. Represents the surface height at location i.
- $\bar{I}$ is the mean height value across all N data points.

### 2.9. T Test

The two-sample *t*-test is a statistical hypothesis test applied to evaluate whether the means of two independent groups differ significantly. It is commonly used to compare two samples and determine if the difference in their means reflects a true underlying effect or is merely attributable to random variation.

The test begins with the null hypothesis (H_0_), which states that there is no significant difference between the means of the two groups, implying that any observed difference arises from chance. In contrast, the alternative hypothesis (H_1_ or H_a_) posits that there is a significant difference between the group means.

The test statistic for a two-sample *t*-test is calculated using the following equation:

(11) $$t=\;\frac{\bar{x}-\;\bar{y}}{\sqrt{\frac{s_{x}^{2}}{n}+\;\frac{s_{y}^{2}}{m}}},$$

where

- $\bar{x},\;\bar{y}\;$ is the sample mean of groups X and Y, respectively;
- $s_{x}^{2}$, $s_{y}^{2}$ is the standard deviation of the two groups;
- n, m is the sample size of the two groups [38,39].

### 2.10. Regions of Interest (ROIs)

Regions of interest (ROIs) were analyzed across three disinfection groups (alcohol, detergent foam, and UVC) in relation to three quantitative descriptors: defect area fraction, anisotropy ratio, and RMS roughness. Performance outcomes were summarized in Table 1 and heat maps, where green indicated the most favorable results, yellow represented intermediate performance, and red denoted the poorest outcomes for each feature. In parallel, boxplots were used to illustrate the distribution of feature values across disinfection methods, highlighting variability and consistency.

Defect area fraction measures the proportion of the surface covered by cracks or holes. Higher values indicate more pronounced surface damage, which can reduce mechanical integrity by acting as stress concentrators that facilitate crack initiation and propagation. Conversely, lower values suggest fewer imperfections and, therefore, a greater likelihood of preserving the original mechanical strength of the material.

Anisotropy ratio reflects the degree of alignment in the carbon structure, which is often correlated with material hardness. A higher anisotropy ratio can enhance hardness and wear resistance but may also reduce ductility, making the material more susceptible to brittle fracture under sudden loads. Lower anisotropy values generally indicate a more isotropic structure, which can improve ductility at the expense of hardness.

RMS roughness quantifies surface height variations and is directly linked to surface smoothness. Rougher surfaces can accelerate fatigue failure by increasing stress concentrations but may improve adhesion in coating applications. Smoother surfaces, with lower RMS roughness values, are generally advantageous for minimizing crack initiation sites and extending fatigue life.

When considered together, these descriptors provide a balanced assessment of the trade-offs introduced by different disinfection methods. An optimal method would minimize defect area fraction, maintain an anisotropy ratio close to the original material’s optimal value for its intended application, and limit changes in surface roughness to avoid compromising fatigue resistance.

### 2.11. Data Visualization

To validate the surface morphology findings obtained from SEM image processing, elemental analysis was performed using energy-dispersive X-ray spectroscopy (EDX). The analysis focused on quantifying the relative concentrations of carbon (C), nitrogen (N), and oxygen (O), as these elements are directly associated with the chemical stability and oxidation state of ABS polymer surfaces following disinfection.

Twelve ABS specimens (diameter ≈ 4 mm, thickness ≈ 3 mm) previously subjected to the disinfection protocols—control (no sterilization), alcohol (70% ethanol), detergent foam, and UVC irradiation—were analyzed by EDX. For each sterilization condition, three independent samples (3 samples) were examined, yielding a total of 12 specimens. All samples were mounted on standard SEM stubs and sputter-coated with a thin layer of gold to minimize charging effects during measurement, while ensuring that the coating thickness did not interfere with elemental detection.

EDX analysis was conducted using an integrated detector coupled with the SEM instrument. Operating conditions were standardized to ensure reproducibility across samples:

- Acceleration voltage: 20 kV;
- Working distance: 11 mm;
- Spot size: 30;
- Collection time per spectrum: 60 s.

Spectra were collected from multiple regions per sample to account for local surface variability, with data averaged to generate representative elemental profiles.

#### Data Acquisition and Processing

For each sample, the relative atomic percentages of C, N, and O were quantified using the instrument’s built-in spectral deconvolution software. The average values from three replicates per group were calculated to represent the elemental composition of each disinfection treatment.

## 3. Results

The three samples were analyzed based on three structural features: Defect Area Fraction, Anisotropy Ratio, and RMS Roughness. For each SEM image, five concentric circular regions of interest (ROIs) with nominal diameters of 4, 6, 8, 10, and 12 cm were extracted, all centered on the geometric midpoint of the sample.

### 3.1. Sample 1

**Figure 3:**
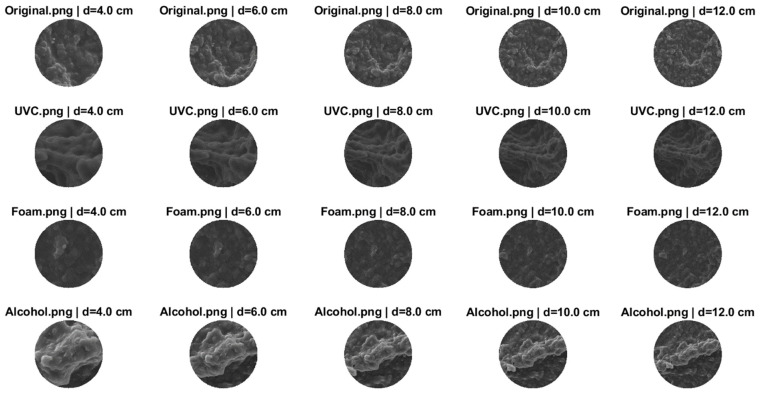
SEM Circular Segments (4–12 cm Diameters) of Original and Disinfected Samples (UVC, Detergent foam, Alcohol).

As shown in Figure 3, the five concentric circular regions of interest (ROIs) with nominal diameters of 4, 6, 8, 10, and 12 cm were extracted from each SEM image after applying the magnification, centered on the geometric center.

To statistically evaluate the impact of disinfection on the surface properties of acrylonitrile–butadiene–styrene (ABS) specimens, a series of two-sample *t*-tests were applied. The control group, which did not undergo any disinfection treatment and thus represented the baseline condition of untreated ABS surfaces, was systematically compared against each of the three disinfection methods under investigation: alcohol, detergent foam, and UVC irradiation. This approach allowed for the determination of whether the observed differences in the measured surface descriptors between the untreated control and the treated groups were statistically significant, thereby providing a rigorous basis for assessing the extent to which each disinfection method alters the microstructural characteristics of ABS surfaces.

Defect area fraction was lowest for detergent foam (0.47966), suggesting this method produced the least cracking or surface damage, which could help preserve the original material’s strength. Alcohol treatment resulted in the highest defect fraction (0.52247), implying a greater density of cracks and holes that could act as initiation points for fracture under load. UVC occupied an intermediate position, indicating moderate preservation of surface integrity.

Anisotropy ratio was highest for UVC (0.013162), suggesting a greater degree of carbon structure alignment, which may enhance hardness and wear resistance. However, excessive alignment may also reduce ductility. Alcohol treatment yielded the lowest anisotropy ratio (0.0019564), indicating reduced alignment and potentially greater ductility, albeit at the expense of hardness. Detergent foam again occupied a middle ground, implying a balance between hardness and ductility.

RMS roughness revealed that UVC produced the smoothest surface (9.54 × 10^−6^), essentially flat, which is advantageous for reducing stress concentrators and improving fatigue resistance. Detergent foam generated the roughest surface (0.055163), which could promote crack initiation during cyclic loading, although in some contexts it may improve coating adhesion. Alcohol fell between the two, maintaining a moderately smooth finish.

The full set of observations is summarized in Table 2.

#### 3.1.1. Heat Map

**Figure 4:**
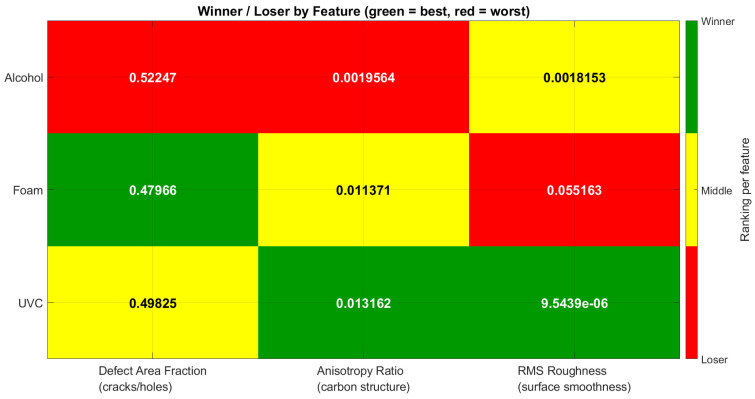
Winner/Loser Heatmap for Surface Defects, Carbon Structure, and Roughness After Disinfection.

The heat map in Figure 4 provides a visual ranking of three disinfection methods—Alcohol, Detergent foam, and UVC—across three key surface-related features: Defect Area Fraction (cracks/holes), Anisotropy Ratio (carbon structure), and RMS Roughness (surface smoothness). Green cells indicate the best performer for a given feature, yellow marks an intermediate position, and red identifies the poorest performer.

##### Defect Area Fraction (Cracks/Holes)

- Best (Green): Detergent foam (0.47966)—Lowest fraction of surface defects, suggesting the best preservation of surface integrity.
- Worst (Red): Alcohol (0.52247)—Highest defect fraction, indicating the most cracking and potential weakening of the material.
- Middle (Yellow): UVC (0.49825)—Moderate preservation of surface quality.

Mechanical implication: Detergent foam treatment is most favorable in terms of limiting surface damage, which could help maintain original strength. Alcohol treatment, by contrast, could accelerate crack initiation and reduce service life.

##### Anisotropy Ratio (Carbon Structure Alignment)

- Best (Green): UVC (0.013162)—Highest structural alignment, potentially leading to increased hardness and wear resistance, though with a possible trade-off in ductility.
- Worst (Red): Alcohol (0.0019564)—Lowest anisotropy ratio, indicating reduced hardness and potentially increased ductility but lower wear resistance.
- Middle (Yellow): Detergent foam (0.011371)—Balanced alignment, possibly retaining some hardness without overly reducing ductility.

Mechanical implication: UVC enhances alignment, which could strengthen the material’s surface properties, while Alcohol treatment appears to compromise hardness significantly.

##### RMS Roughness (Surface Smoothness)

- Best (Green): UVC (9.54 × 10^−6^)—Extremely smooth surface, minimizing stress concentrators and likely improving fatigue resistance.
- Worst (Red): Detergent foam (0.055163)—Roughest surface, which may increase crack initiation under cyclic loading but could improve coating adhesion in some cases.
- Middle (Yellow): Alcohol (0.0018153)—Moderately smooth surface.

Mechanical implication: UVC’s smoothness is advantageous for applications where fatigue life is critical. Detergent foam’s roughness, while mechanically risky for fatigue, may be useful in processes where surface adhesion is desirable.

##### Overall Assessment

- UVC: Strength in anisotropy and smoothness, moderate defect control. Likely to produce a harder, smoother surface but may slightly reduce ductility.
- Detergent foam: Excellent defect control, moderate anisotropy, but poorest smoothness. May retain material strength but with higher fatigue crack risk due to roughness.
- Alcohol: Weakest overall—highest defects, lowest anisotropy, only moderate smoothness—suggesting both structural and mechanical disadvantages. The observations can be summarized in Table 3.

### 3.2. Sample 2

**Figure 5:**
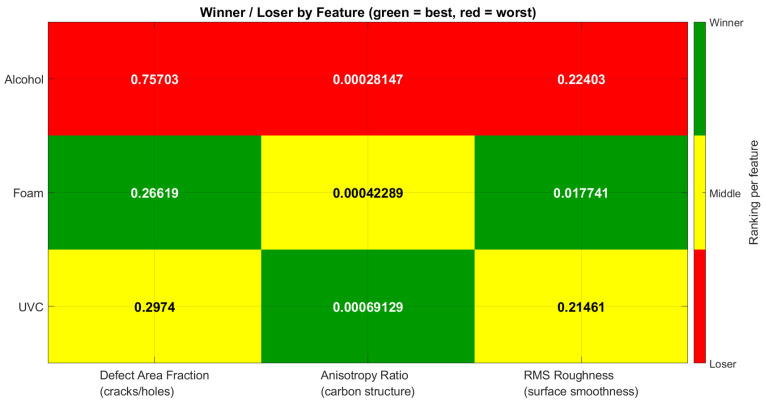
Comparative Heat Map of Disinfection Methods Based on Feature Rankings (Defect Area Fraction, Anisotropy Ratio, RMS Roughness.

As shown in the heatmap in Figure 5, Detergent foam performed the best across all three structural features. For the defect area fraction, Detergent foam showed the lowest level of cracks and holes, followed by UVC, while Alcohol had the highest defects. Similarly, in terms of anisotropy ratio, Detergent foam again achieved the best carbon structural alignment, with UVC showing moderate performance and Alcohol remaining the weakest. Finally, for surface smoothness (RMS roughness), Detergent foam produced the smoothest surface, UVC was less effective, and Alcohol showed the roughest texture. Overall, Detergent foam consistently outperformed the other disinfection methods in this sample.

### 3.3. Sample 3

**Figure 6:**
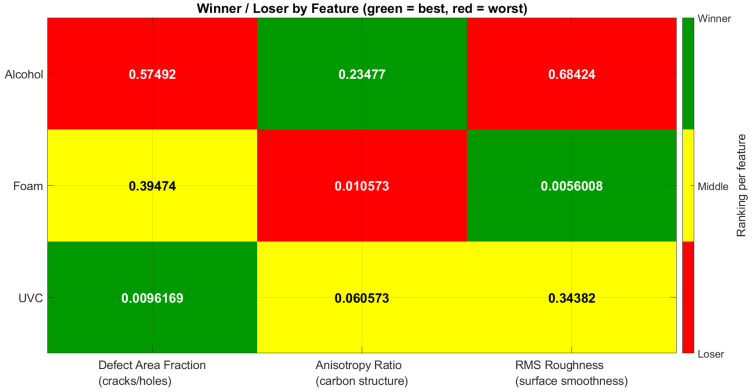
Comparative Heat Map of Disinfection Methods by Microstructural Features (Sample 3).

As shown in the heatmap in Figure 6, UVC showed the best performance in two of the three structural features. For the defect area fraction, UVC achieved the lowest level of cracks and holes, followed by Detergent foam, while Alcohol was the worst. In terms of anisotropy ratio, UVC again provided the best carbon structural alignment, Alcohol showed moderate performance, and Detergent foam performed the weakest. However, when looking at surface smoothness (RMS roughness), Detergent foam produced the smoothest surface, UVC was moderate, and Alcohol had the roughest texture. Overall, UVC was superior in structural integrity, while Detergent foam excelled only in surface smoothness.

### 3.4. Combined Heat Map

**Figure 7:**
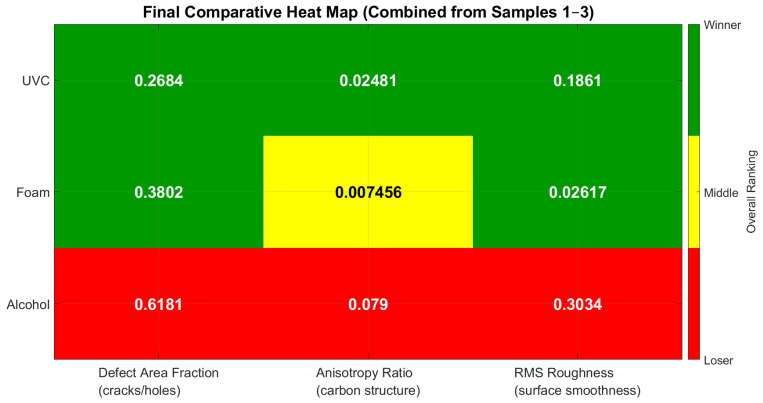
Final Comparative Heat Map of Disinfection Methods by Microstructural Features (Combined Results from Samples 1–3).

**Figure 8:**
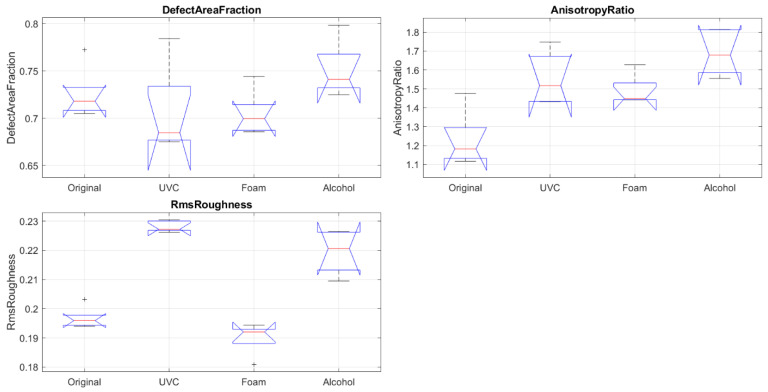
Boxplots of SEM-Derived Features (Defect Area Fraction, Anisotropy Ratio, and RMS Roughness) for Different Disinfection Methods Compared to the Original. As shown in Figure 8, the “+” symbol indicates the outliers, the red line represents the mean value, and the blue line denotes the baseline reference for the measured feature.

The combined heat map and box plot, shown in Figure 7 and Figure 8, respectively, summarize the performance of three disinfection methods (alcohol, detergent foam, and UVC) across three critical features: defect area fraction (cracks/holes), anisotropy ratio (carbon structure), and RMS roughness (surface smoothness). Each feature was evaluated statistically across three independent samples, and the rankings (best, intermediate, worst) were consolidated based on majority agreement, with corresponding average *p*-values annotated in the heat map.

Based on the consolidated results, UVC irradiation was identified as the most effective disinfection method, offering the best balance between minimizing surface defects, preserving carbon structure, and maintaining smoothness. Detergent foam provided acceptable but less consistent performance, while alcohol disinfection should be avoided as it caused the greatest structural and surface degradation.

### 3.5. Energy Dispersive X-Ray Spectroscopy (EDX or EDS)

The EDX analysis was performed to evaluate the surface chemical composition of the material before sterilization (Original) and after applying three sterilization methods: Foam, UVC, and Alcohol. The measured elemental distributions included Carbon (C), Nitrogen (N), and Oxygen (O), which reflect different surface and structural characteristics:

- Carbon (C): Indicates structural integrity of the surface. Values closer to the original suggest better preservation of the material.
- Nitrogen (N): Associated with surface chemistry stability. Higher or stable values indicate more balanced surface interactions and smoother chemistry.
- Oxygen (O): Correlated with oxidation and defects. An increase in oxygen content suggests higher surface defects, while a reduction implies fewer oxidation-related changes.

SEM revealed the extent of surface damage—defects, roughness, and carbon structural disruption—while EDX clarified the chemical basis of these changes through carbon (structural integrity), nitrogen (surface stability), and oxygen (oxidation). Both techniques produced the same ranking of sterilization methods.

**Figure 9:**
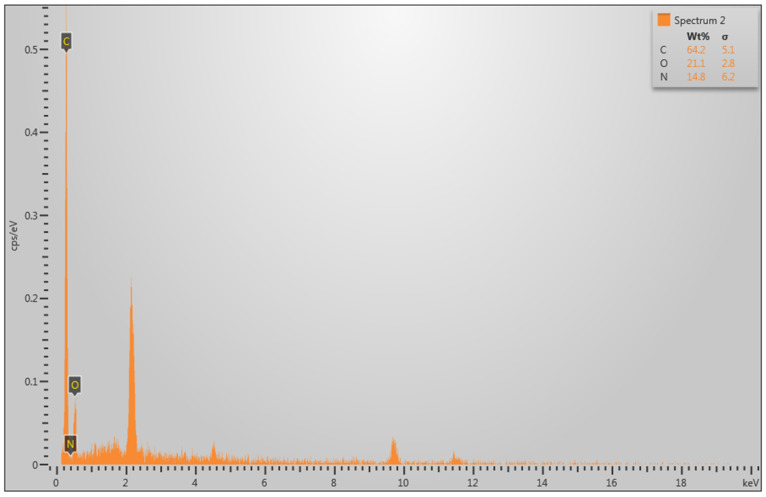
EDX Spectrum of the Original (before sterilization) Showing Carbon, Nitrogen, and Oxygen Distribution.

The EDX spectrum shows three dominant peaks corresponding to Carbon (C), Oxygen (O), and Nitrogen (N). These elements represent the main surface composition of the material before applying any sterilization method. From the quantitative results provided in the spectrum, Carbon (C): 64.2; Oxygen (O): 21.1; and Nitrogen (N): 14.8 as shown in Figure 9.

Following the acquisition of all EDX spectra for the detected elements, the results are summarized in Table 4.

Foam sterilization produced the highest Carbon content (~69.7 vs. 64.1 original), suggesting significant alteration of structural integrity. Nitrogen decreased compared to the original (~19.6 vs. 22.0), indicating less stable surface chemistry, while Oxygen decreased substantially (~10.7 vs. 13.9), implying reduced oxidation. UVC sterilization showed moderately increased Carbon (~68.2), while Nitrogen remained close to the original (~24.2 vs. 22.0), reflecting good surface stability. Oxygen decreased sharply (~7.6), indicating the lowest oxidation among all methods. Alcohol sterilization also increased Carbon (~68.8) but caused a marked reduction in Nitrogen (~17.3), highlighting disturbed chemistry. Oxygen remained similar to the original (~13.9), showing no improvement in oxidation control.

**Figure 10:**
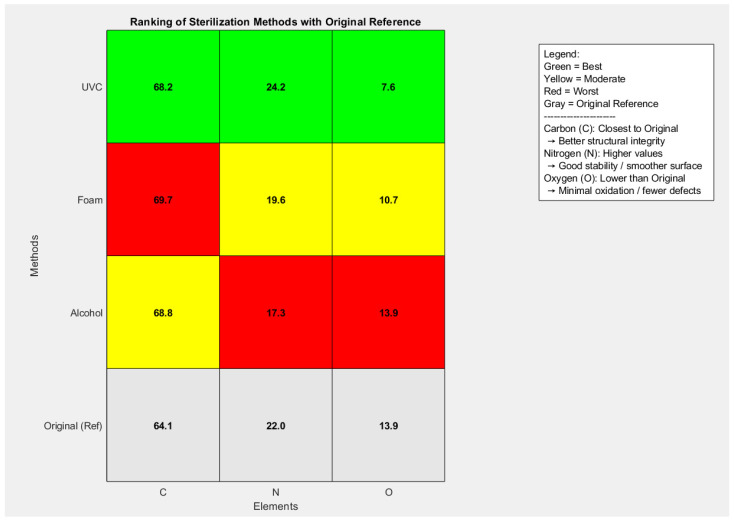
Heatmap Ranking of Sterilization Methods Compared to Original Reference (C, N, O Composition).

The heatmap visualization clearly demonstrated that UVC achieved the best balance, minimizing oxidation while maintaining nitrogen stability, as shown in Figure 10.

#### Statistical Evaluation (*t*-Test and *p*-Values)

*t*-tests were conducted comparing each sterilization method to the Original for every element. The resulting *p*-value heatmap highlighted significant differences: For Carbon (C) and Nitrogen (N), higher *p*-values indicate similarity to the original. For Oxygen (O), lower *p*-values are desirable, reflecting reduced oxidation.

The results showed that UVC sterilization consistently yielded the most favorable statistical profile: high *p*-values for Nitrogen (0.554, closest to original stability) and low *p*-values for Oxygen (0.166, confirming significant oxidation reduction). Foam sterilization demonstrated reduced oxidation (*p* = 0.350 for O) but significant deviations in Carbon (*p* = 0.019), highlighting structural alteration. Alcohol sterilization had the weakest performance overall, with low *p*-values for Nitrogen (0.211) and very high for Oxygen (0.988), confirming poor control of surface chemistry and oxidation. By integrating the elemental averages and statistical results, UVC sterilization ranked best, providing the lowest oxidation, stable nitrogen chemistry, and acceptable carbon deviation. Foam sterilization ranked second, effective in reducing oxidation but compromising both carbon structure and nitrogen stability. Alcohol sterilization ranked last, as it destabilized nitrogen and failed to improve oxidation compared to the original surface.

## 4. Discussion

### 4.1. SEM Analysis of Sterilization Methods

The SEM-derived heat map provided a quantitative assessment of surface morphology based on three descriptors: defect area fraction (cracks/voids), anisotropy ratio (carbon structural alignment), and RMS roughness (surface smoothness). The statistical results demonstrated clear differences among the sterilization methods. UVC sterilization consistently produced the most stable morphological profile, showing minimal deviation from the untreated surface across all three parameters, in agreement with its favorable elemental statistics (high *p*-value for nitrogen, 0.554, and low *p*-value for oxygen, 0.166). Foam sterilization exhibited an intermediate response; although some surface features remained comparable to UVC, the anisotropy ratio and carbon-related metrics showed significant deviation (e.g., carbon *p* = 0.019), indicating structural alteration despite reduced oxidation (oxygen *p* = 0.350). In contrast, alcohol sterilization showed the highest surface disruption, reflected by greater defect density, disturbed carbon structures, and increased roughness, consistent with its unfavorable elemental profile (nitrogen *p* = 0.211; oxygen *p* = 0.988). Taken together, the SEM and EDX results converge to the same ranking: UVC provides the most stable and least oxidized surface, foam yields moderate stability with notable carbon perturbation, and alcohol causes the most pronounced microstructural and chemical degradation.

### 4.2. EDX Analysis as a Validation Tool

While SEM highlights surface morphology, it does not directly account for chemical alterations. For this reason, EDX elemental analysis was employed as a complementary validation technique. The elemental distribution (C, N, O) confirmed the morphological patterns observed in SEM. UVC-treated samples showed elemental stability (C: 68.2, N: 24.2, O: 7.6), mirroring the SEM finding of preserved structural integrity. Foam treatment, which SEM identified as moderately disruptive, likewise showed reduced nitrogen (19.6) and elevated carbon (69.7), supporting the interpretation of altered carbon structure and reduced stability. Alcohol sterilization produced the most pronounced elemental imbalance (C: 68.8, N: 17.3, O: 13.9), aligning with SEM evidence of significant surface roughness and defects. The elevated oxygen content further indicated surface oxidation, confirming the destabilizing effect detected morphologically.

### 4.3. Cross-Validation of SEM by EDX

The convergence of SEM and EDX results underscores their complementarity. SEM quantified the degree of surface damage through image-based processing, while EDX verified these findings chemically by linking morphological irregularities to elemental depletion and oxidative changes. The parallel outcomes strengthen the validity of SEM image processing, demonstrating that observed surface alterations are not artifacts of imaging but are underpinned by measurable chemical modifications.

The SEM analysis in Section 4.1 describes how the surface appears—including defect formation, roughness changes, and disturbances in carbon structural alignment—whereas the EDX analysis in Section 4.2 explains what the surface is chemically composed of, through the distribution of carbon (C), nitrogen (N), and oxygen (O). These elemental indicators provide meaningful insight into surface stability: carbon reflects structural integrity, where values close to the untreated sample indicate better preservation of the material; nitrogen represents surface chemistry stability, where stable or higher values suggest balanced interactions and smoother chemical behavior; and oxygen is directly linked to oxidation, where increases indicate defect formation and degradation, while lower levels imply reduced oxidative damage. Although SEM and EDX measure different aspects of the surface, their results converge clearly, showing the same ranking of sterilization methods and the same pattern of material degradation.

UVC sterilization demonstrated the most stable behavior. SEM revealed minimal defects, low roughness, and preserved carbon alignment, and EDX confirmed this chemically through stable carbon and nitrogen levels with minimal oxygen increase—indicating low oxidation. Foam sterilization produced an intermediate effect: SEM showed moderate disruption in carbon structure, and EDX reflected this through reduced nitrogen and elevated carbon, with oxygen levels indicating moderate oxidation. Alcohol sterilization caused the most severe damage. SEM detected increased roughness, higher defect density, and pronounced structural disturbance, and EDX confirmed this through reduced nitrogen and elevated oxygen, indicating significant oxidation and chemical instability.

Thus, the morphological changes observed by SEM are directly supported by the elemental signatures measured by EDX. The strong agreement between structural and chemical evidence reinforces the reliability of the results and validates the ranking of the sterilization methods. This is referred to as convergence, which represents a stronger form of agreement than simple correlation.

### 4.4. Comparative Ranking

The combined analyses establish a robust ranking of sterilization efficacy: UVC sterilization consistently emerges as the most favorable method, preserving both surface morphology and elemental balance. Foam sterilization produces moderate alterations, evident in both anisotropy and nitrogen depletion, reflecting partial but controlled disruption. Alcohol sterilization is the least favorable, as confirmed by both morphological and chemical evidence of significant degradation and oxidation.

In summary, EDX serves as a reliable validation tool for SEM image processing outcomes, providing chemical confirmation of the morphological changes quantified by SEM. Together, these techniques offer a comprehensive framework for evaluating sterilization effects, with UVC emerging as the superior method due to its minimal structural and elemental impact.

## 5. Conclusions

This study presents the first systematic evaluation of acrylonitrile–butadiene–styrene (ABS) surfaces subjected to three clinically relevant sterilization methods: UVC irradiation, detergent disinfection foam, and 70% alcohol. By combining a novel SEM image processing approach with traditional EDX elemental validation, both morphological and chemical alterations were comprehensively assessed across 12 specimens. SEM analysis revealed clear differences in surface integrity among treatments, with UVC consistently producing the lowest defect fractions, balanced anisotropy, and smoother surfaces. Detergent foam showed intermediate effects, with significant anisotropy alterations but defect and roughness values largely comparable to UVC. Alcohol, in contrast, caused the greatest deterioration, characterized by higher defect density, disrupted carbon structures, and rougher surfaces.

EDX analysis, focusing on carbon (C), nitrogen (N), and oxygen (O), corroborated the SEM findings. UVC-treated samples maintained elemental stability, while foam-treated samples exhibited reduced nitrogen and elevated carbon, consistent with the observed structural changes. Alcohol sterilization produced the most pronounced elemental imbalance, with oxygen enrichment and nitrogen depletion indicative of oxidation and instability. The strong convergence between SEM and EDX results underscores the reliability of the proposed SEM image processing technique as a novel method for surface characterization, validated against a well-established analytical standard.

Taken together, the integrated results establish a clear ranking of sterilization efficacy: UVC emerges as the most effective method for preserving both surface morphology and elemental balance, detergent foam offers a reliable and clinically acceptable alternative, and alcohol disinfection is not recommended due to its detrimental effects on surface integrity and composition. Beyond these findings, this study demonstrates the value of SEM image processing, validated by EDX, as a reproducible and decision-oriented framework for assessing material responses to sterilization.

Future studies are recommended to investigate the interaction between sterilization methods and microbial behavior on ABS surfaces, including bacterial adhesion and biofilm formation. Integrating microbiological assays with surface morphological and elemental analysis would provide a more comprehensive understanding of how sterilization efficacy correlates with material compatibility and long-term performance.

Future studies are encouraged to integrate additional surface characterization techniques such as AFM or confocal microscopy to further validate the computational measurements extracted from SEM images. Moreover, the full post-processed surface patterns and feature-extraction maps will be included in subsequent work to enhance visual transparency and provide deeper insight into the spatial distribution of defects beyond the detected ROIs presented in this study.

## Figures and Tables

**Table 1:** Understanding the Features and What Higher/Lower Means.

Feature	Meaning	Higher Value Implies	Lower Value Implies
Defect Area Fraction	Fraction of surface area with cracks/holes	More surface defects → potential reduction in mechanical integrity	Fewer defects → potentially better mechanical strength
Anisotropy Ratio	Degree of carbon structure alignment; linked to hardness	More alignment → higher hardness, but often less ductility	Less alignment → lower hardness, possibly more ductility
RMS Roughness	Surface height variation (nm/µm range)	Rougher surface → can initiate cracks; may aid bonding in coatings	Smoother surface → fewer stress concentrators, better fatigue resistance

**Table 2:** Quantitative Surface Descriptors (Defect Area Fraction, Anisotropy Ratio, RMS Roughness) for ABS under Disinfection Treatments.

Method	Defect Area Fraction	Anisotropy Ratio	RMS Roughness
UVC	0.49825	0.013162	9.54 × 10^−6^
Detergent foam	0.47966	0.011371	0.055163
Alcohol	0.52247	0.0019564	0.0018153

**Table 3:** Comparative Mechanical Impact of Disinfection Methods Based on Surface Feature Analysis.

Method	Strengths	Weaknesses	Likely Mechanical Impact
UVC	Best smoothness, highest anisotropy	Moderate defects	Harder, smoother, possible ductility reduction
Detergent foam	Lowest defects, balanced anisotropy	Roughest surface	Strong but higher fatigue crack risk
Alcohol	None dominant	Most defects, lowest anisotropy	Structurally weaker, reduced hardness

**Table 4:** Elemental Composition (C, N, O) of the Material Surface Before and After Different Sterilization Methods.

Before Sterilization			Foam			UVC			Alcohol		
C	N	O	C	N	O	C	N	O	C	N	O
62	27.8	16.8	70.9	20.4	8.7	69	22.4	8.6	67.9	17.9	14.2
66.1	17.1	10.1	70.2	21.6	8	64.2	23.3	12.5	68	17	15
64.2	21.1	14.8	68	16.7	15.3	71.4	26.9	1.7	70.5	17.1	12.4

## Data Availability

The original contributions presented in this study are included in the article. Further inquiries can be directed to the corresponding author.
